# Supplementary material for: Integration of 3D Hydrodynamic Focused Microreactor with Microfluidic Chemiluminescence Sensing for Online Synthesis and Catalytical Characterization of Gold Nanoparticles
Source: Sensors (Basel). 2021 Mar 25;21(7):2290. doi: 10.3390/s21072290 (PMC8036713; doi:10.3390/s21072290)
Supplement: Supplementary file 1 [file sensors-21-02290-s001.pdf]

Supporting information

Integration of 3D hydrodynamic focused microreactor with microfluidic chemiluminescence sensing for online synthesis and catalytical characterization of gold nanoparticles

Yanwei Wang and Michael Seidel*

Institute of Hydrochemistry, Chair of Analytical Chemistry and Water Chemistry, Technical University of Munich, Elisabeth-Winterhalter-Weg 6, 81377 Munich, Germany. Yanwei.wang@tum.de

* Correspondence: E-mail: Michael.Seidel@mytum.de, Tel: +49 89 2180 78252, Fax: +49 89 2180 78255

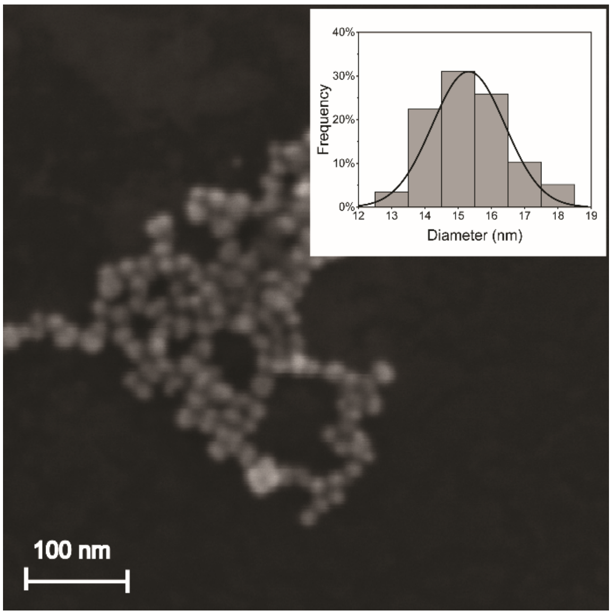

Figure S 1. SEM images of synthesized AuNPs with optimal synthesis conditions. Histograms with the respective particle size distributions and the percentage are included as inset.
